# Machine learning approaches to identify influential factors associated with hypertension and prehypertension among rural adults in Bangladesh

**DOI:** 10.1177/22799036261467444

**Published:** 2026-08-25

**Authors:** Md Zahidul Islam, Mohammad Rocky Khan Chowdhury, Zarin Raihana, Farzana Akhter Bornee, Farah Naz Rahman, Shanta Rani Biswas, Mamunur Rashid

**Affiliations:** 1 Institute of Biological Sciences, 665056 University of Rajshahi, Rajshahi, Bangladesh; 2 Department of Public Health, First Capital University of Bangladesh, Chuadanga, Bangladesh; 3 Department of Population Science, Jatiya Kabi Kazi Nazrul Islam University, Mymensingh, Bangladesh; 4 Department of Epidemiology and Preventive Medicine, School of Public Health and Preventive Medicine, 2541 Monash University, Melbourne, VIC, Australia; 5 Department of Clinical Psychology, Faculty of Biological Sciences, 665056 University of Rajshahi, Rajshahi, Bangladesh; 6 Department of Pediatrics, Bangladesh Medical University, Dhaka, Bangladesh; 7 Department of English, 54493 University of Chittagong, Chittagong, Bangladesh; 8 Unit of Public Health Science, Faculty of Health and Occupational Studies, 3485 University of Gävle, Gävle, Sweden

**Keywords:** hypertension, prehypertension, machine learning, rural area, Bangladesh

## Abstract

**Background:**

Hypertension poses a significant public health challenge in Bangladesh, particularly among rural populations with limited access to healthcare. Thus, this study aimed to identify the influential factors associated with hypertension and prehypertension in rural Bangladesh using sophisticated machine learning (ML) methods.

**Methods:**

A total of 1603 respondents were selected in this study using a multistage random sampling from a cross-sectional survey. Five commonly used sophisticated ML algorithms were used. The predictive performance of these models was evaluated using standard validation metrics. Influential variables were identified and ranked using SHapley Additive exPlanations (SHAP) technique.

**Results:**

The prevalence of hypertension and prehypertension was 30.9% and 40.8%, respectively. The XGB model outperformed other ML models in predicting hypertension (accuracy: 74.3%, ROC: 75.8%), while the LR model was better at predicting prehypertension (accuracy: 59.2%, ROC: 54.1%). Top factors in predicting hypertension were older age, being overweight or obese, having a past or no smoking history, reporting no chronic disease, and having a family history of hypertension, whereas top factors for pre-hypertension were current smoking status, absence of cardiovascular disease, being in a younger or middle-aged group, having no family history of hypertension, and current employment.

**Conclusion:**

Three out of ten people in rural areas were hypertensive, while two out of five were prehypertensive. The ML models had the potential to predict hypertension. The current findings highlight an urgent need for strengthened national and regional public health initiatives to improve hypertension detection, awareness, and management in rural Bangladesh.

**Public health significance**

Hypertension is an increasing public health issue in Bangladesh, particularly in rural areas where access to healthcare is limited. The influential factors associated with hypertension and prehypertension have been insufficiently studied in rural areas. Using machine learning, this study identified key factors linked to hypertension and prehypertension, showing that older age, excess body weight, and family history mainly drive hypertension; while smoking and work-related stress are more common among those with prehypertension. These results highlight that early prevention should start with younger, working adults through healthier lifestyle promotion, smoking cessation, and stress management. At the same time, regular screening and weight control programs are essential for older and high-risk groups. By revealing which factors matter most, this research can help public health professionals design more focused, data-driven strategies to prevent high blood pressure and reduce the growing burden of heart disease in Bangladesh.

## Introduction

Hypertension is a serious chronic condition that significantly increases the risk of mortality from cardiovascular diseases. Affecting nearly one in four adults worldwide, it accounts for one in every five deaths globally.^1^ Conversely, prehypertension is also linked to an increased risk of cardiovascular disease and progression to hypertension.^2^ This growing burden of hypertension is unevenly distributed, with low- and middle-income countries, particularly in South Asia, witnessing a significant increase in cases.^1^ The situation in countries like Bangladesh is particularly concerning, highlighting the urgent need for a deeper understanding of the factors associated with hypertension and related conditions.^3,4^ The shifting demographic and epidemiological landscape, especially in Bangladesh, has contributed to a worrisome rise in hypertension and other non-communicable diseases.^5^ Among adults in Bangladesh, the prevalence of hypertension and prehypertension is alarmingly high, affecting approximately 29% and 35% of the population, respectively.^1,6^

The burden of hypertension is particularly pronounced in rural areas, where the prevalence is approximately 25%, and 68.5% of the total population resides.^7^ The rural population in Bangladesh are experiencing an increased incidence of chronic conditions like hypertension, which adversely affect both their health and livelihoods.^4^ Limited access to healthcare services, inadequate media coverage and insufficient information resources further exacerbate the risk of hypertension in these populations.^8^ Additionally, the economic burden of hypertension imposes significant challenges, particularly for rural populations, leading to substantial out-of-pocket expenditures.^8^

Although several studies in Bangladesh have identified various risk factors for hypertension – encompassing demographic, anthropometric, lifestyle, and clinical factors – using national, urban, and rural community-based data, a gap remains in understanding the influential factors specifically associated with prehypertension in rural communities.^3,5,9–15^ Prior to 2019, only a few community-based studies in Bangladesh assessed risk factors for hypertension and prehypertension, and most relied on traditional regression models.^14,16–18^ Consistency in monitoring non-communicable disease patterns over time in these settings is crucial, given the growing demand driven by worsening trends. Also, traditional regression models face limitations when dealing with real-world data that exhibit complex, high-dimensional, and non-linear patterns, often resulting in low precision at the patient level.^19^ In contrast, ML models can automatically capture complex, nonlinear relationships and interactions among a large number of variables without strict assumptions. Further, ML models outperformed traditional regression models in predicting outcomes in different public health areas.^20–22^

Additionally, machine learning (ML) approaches remain underutilized in Bangladesh for identifying influential factors associated with hypertension, particularly in studies using nationally representative data.^23–25^ Previous studies have mainly identified factors such as age, BMI, education, and wealth index as key predictors of hypertension.^23,25–28^ Moreover, to the best of our knowledge, no prior study has specifically explored the influential factors associated with both hypertension and prehypertension in rural populations in Bangladesh using ML algorithms. This study aims to bridge this gap through identifying the potential influential factors of hypertension and prehypertension in rural Bangladesh by employing several ML algorithms and assessing the prediction performances. In doing so, we aim to enhance our understanding of these conditions in rural areas, enabling early detection, diagnosis, and effective management to improve public health outcomes.

## Methods

### Survey management

The recruitment process for field personnel and the training workshop (Supplementary text S1), the data collection instruments (Supplementary text S2), the pilot survey (Supplementary text S3), and quality assurance (Supplementary text S4) were presented in the **Supplementary materials**.

### Study design, setting, and population

This cross-sectional study was conducted from 1st August to 30th November 2021, employing a multistage random sampling method to recruit participants from 18 villages in a randomly selected district in Bangladesh. Information was collected from 1603 participants in this study.

### Sample size calculation/power analysis

The study collected blood pressure (BP) data from residents of rural villages, calculating the sample size using the formula: n = Z^2^_1−α/2_ × p (1- p) ÷ d^2^. In this formula, n is the sample size, p represents the prevalence of hypertension in rural Bangladesh (around 27% according to a recent study), and d refers to the desired precision (set at 3%).^29^ Using a standard normal deviate value (Z_1−α/2_) of 1.96 for a 95% confidence level, the initial sample size was calculated as 841 participants. After adjusting for a design effect of 1.75 due to the multi-stage nature of the study, the final sample size was increased to 1472.^30,31^ However, an additional 131 participants were recruited in the actual study to adjust the non-response rate.

### Sampling technique

Geographically, Bangladesh is divided into eight administrative regions, known as divisions, including Barisal, Chittagong, Dhaka, Khulna, Mymensingh, Rajshahi, Rangpur, and Sylhet (the first layer of administration). At the initial stage, a Khulna division was randomly selected, and then a district, Jhenaidah (the second administrative layer within a division), was randomly selected from this division (among 10 districts). Thereafter, an Upazila – a sub-unit of a district namely Jhenaidah sadar (third layer of administrative region within a district) was randomly selected from the recruited district (among 6 Upazilas). Finally, a Union Parishad – a sub-unit of an Upazila, namely Naldanga (the last administrative layer) - was randomly selected from Jhenaidah Sadar (among 14 Unions). Participants were recruited from 18 of the Naldanga union’s 22 villages. An equal proportion of participants was maintained throughout recruitment, with a minimum of 90 participants recruited from each village. Respondents from the remaining four villages were not contacted to meet the required target sample size.

This study followed the ‘Kish Grid’ method to select a household and study participants.^32^ This method permitted interviewing only one member from the selected household. The interviewers chose the first household to enroll in the study by identifying the one closest to the center point of the Union Parishad. Then, applying inclusion criteria (the adults aged 18 years and above who reside in the recruited household within the selected villages and provided written consent) as reported by a previous study,^33^ a household member was interviewed. Pregnant women, mentally disabled and people who had surgery in the last three months were excluded from reporting. Further, if a chosen household member declined to participate or the household was inaccessible, it was considered a refusal, and the subsequent household would be approached. During the data collection phase, efforts were made to maintain proportional representation of sex and age groups by ensuring an approximately equal number of respondents in each sex category (male and female) and within each age stratum (young adults, adults, middle-aged adults, and old adults). The sampling technique was presented in Supplementary Figure S1.

### Outcome variables

The outcome variables in this study were hypertension and prehypertension. The current study followed the Seventh report of the Joint National Committee on Prevention on Detection, Evaluation, and Treatment of High Blood Pressure (JNC 7) BP classification system to identify hypertensive patients (systolic blood pressure (SBP) of ≥140 mmHg and/or average diastolic blood pressure (DBP) of ≥ 90 mmHg).^34^ A participant was considered hypertensive (already diagnosed), if they presented with a documented diagnosis of hypertension provided by a registered medical professional, and/or if they were taking antihypertensive medications prescribed by a registered health care professional at the time of data collection.^35^ Participants were unaware and had an average SBP >140 mmHg and/or average DBP > 90 mmHg at the time of data collection and currently taking no treatment with antihypertensive medication was also considered hypertensive. Prehypertension is classified as an average SBP between 120 and 139 mmHg and/or an average DBP of 80 to 89 mmHg.

All anthropometric and BP measurements were conducted by a registered nurse using a calibrated digital monitor (ELITE YE770A) with a standard cuff. The device’s accuracy was routinely verified against a certified reference manometer at multiple pressure points (50–200 mmHg), ensuring readings within ±3 mmHg of the reference standard. Participants were instructed to abstain from coffee and smoking for at least 30 minutes before measurement and to rest for five minutes in a seated position with feet flat and legs uncrossed. BP was measured on the arm free of excess clothing, positioned at heart level, and the same arm was used for all measurements to ensure consistency. Three readings were recorded at 15-minute intervals, and the average of these readings was used as the participant’s final BP value.

### Independent variables

The variables considered in this study were demographic characteristics, which include age, sex of the respondents, educational status, employment status, and marital status; lifestyle factors, such as chewing tobacco, and smoking history; anthropometric characteristics such as body mass index (BMI), and waist-hip ratio; clinical characteristics such as diabetes, cardiovascular disease (CVD), other chronic diseases, and family history of hypertension. Most of the variables in this study were found to be significant in previously published literature.^3,5,11,15,36^ Detailed operational definitions and scale of measurements of independent variables were presented in Supplementary Table S1.

In this study, both outcomes and independent variables were measured concurrently at the time of data collection, reflecting a cross-sectional design. Therefore, the temporal relationship between independent variables and outcomes cannot be definitively established. While some variables, such as age, sex, and family history, are inherently antecedent to hypertension, other factors (e.g., BMI, lifestyle factors, and comorbid conditions) were assessed simultaneously with BP measurements. As a result, the observed associations represent correlations rather than causal relationships.

### Statistical analysis

Baseline characteristics were assessed through descriptive analysis. The relationship between independent and dependent variables was examined using a Chi-square test, with significance considered when a p-value below 0.05 was achieved.

#### Development of the ML models

The ML approaches are highly effective in handling complex, unstructured big data from patient databases. These methods can often detect complex patterns within large datasets comprising numerous types of variables such as binary, categorical, continuous variables, and images, enabling the exploration of interactions and nonlinear relationships that traditional statistical techniques may struggle to explain.^37^ A comprehensive literature review identified several ML algorithms as superior in predicting hypertension, including K-Nearest Neighbour (KNN), Logistic Regression (LR), Random Forest (RF), Support Vector Machine (SVM) and Extreme Gradient Booster (XGB) (Supplementary Table S2).^23–25,38^ These algorithms are capable to select relevant variables after model fitting.^39^

The ML models were developed using 60% of the dataset as a training set, while the remaining 40% was reserved for validation (test set). Each model underwent hyperparameter tuning through a 5-fold cross-validation protocol (Supplementary Table S3). Following this, all trained models were further validated using the 40% validation dataset.

**Figure 1.**
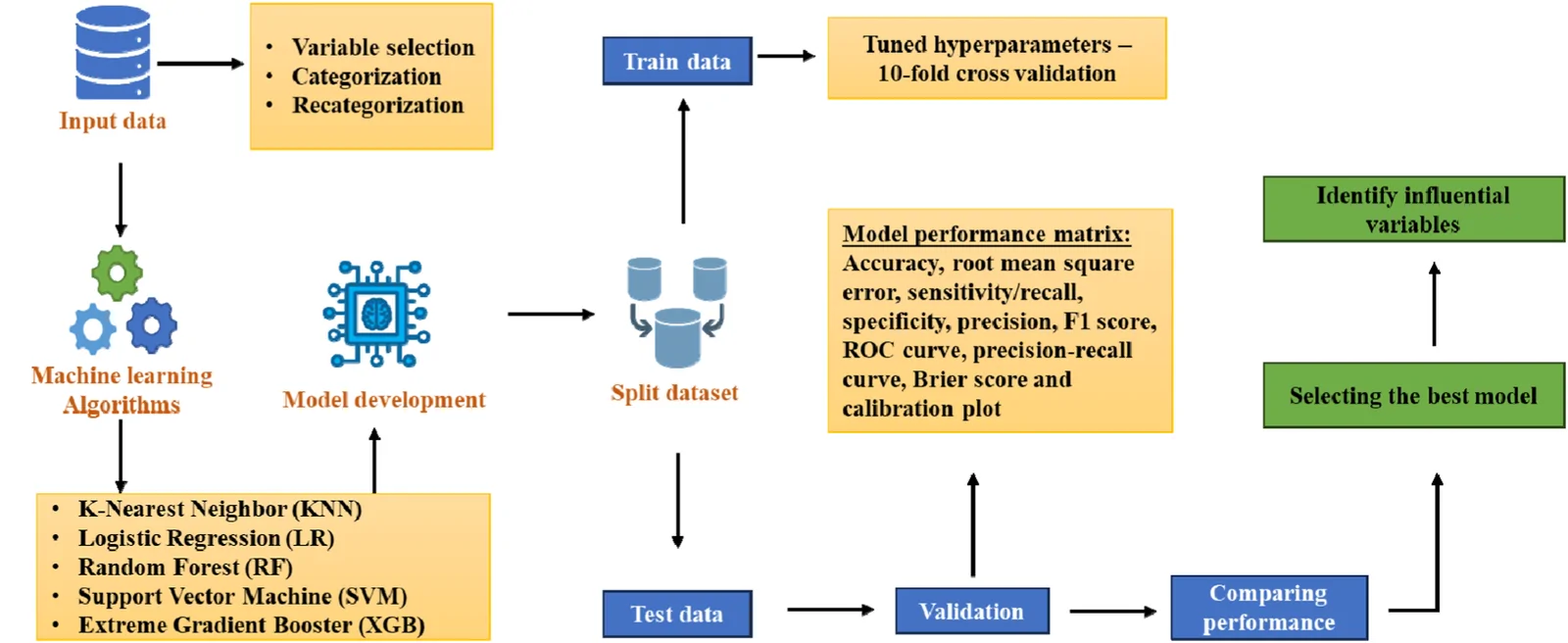
Schematic presentation of selecting the best ML model.

The best-performing ML model was selected based on a comparison of performance metrics for the validation dataset, including accuracy, root mean square error (RMSE), sensitivity/recall, specificity, precision, F1 score, receiver operating characteristic (ROC) curve with 95% Confidence Interval (CI), precision-recall (PR) curve with 95% CI, Brier score and calibration plot (Supplementary Table S4). A schematic representation of the selection process for the best ML model is shown in Figure 1.

#### Interpretability of influential factors in the model

The key factors identified by the best-performing ML algorithm for predicting both hypertension and prehypertension were selected using the SHapley Additive exPlanations (SHAP) technique. The SHAP technique, developed by Lundberg and Lee, incorporates principles from game theory and local explanation methodologies to evaluate the influence of individual features on a model’s decision-making process.^40–42^

All data analysis was carried out using statistical software packages, specifically Stata (version 17), and Python (version 3.9).

## Results

### Background profile of the respondents

Around 26.7% of participants were aged 50 years or older. The distribution between sexes was nearly equal, with 51% were male. Approximately 27.4% of the respondents had no formal education. Around 19.7% of respondents reported smoking habits. Around 56.8% of participants were overweight or obese. Around 8% of respondents had diabetes, and 5.4% had CVD. A family history of hypertension was reported by 27.8% of participants. Symptomatic anxiety was reported in approximately 16% of individuals, and around 18% experienced symptoms of depression. A detailed background profile is provided in Table 1.

**Table 1.** Prevalence of hypertension and prehypertension in rural Bangladesh.

Factors	Frequency (%)	Hypertension		Prehypertension	
		N (%)	p-value	N (%)	p-value
**Age (in years)**					
30 and less	377 (23.5)	51 (13.5)	<0.001	166 (44.0)	0.005
31-50	799 (49.8)	243 (30.4)	​	342 (42.8)	​
50 and above	427 (26.7)	202 (47.3)	​	146 (34.2)	​
**Sex of the respondent**					
Male	818 (51.0)	243 (29.7)	0.275	347 (42.4)	0.177
Female	785 (49.0)	253 (32.2)	​	307 (39.1)	​
**Educational status**					
No formal education	439 (27.4)	165 (37.6)	0.003	169 (38.5)	0.115
Primary	409 (25.5)	125 (30.6)	​	156 (38.1)	​
Secondary	572 (35.7)	156 (27.3)	​	256 (44.8)	​
Higher	183 (11.4)	50 (27.3)	​	73 (39.9)	​
**Employment status**					
Employed or self-employed	714 (44.5)	195 (27.3)	0.008	309 (43.3)	0.193
Housewife	713 (44.5)	234 (32.8)	​	276 (38.7)	​
Retired or student	176 (11.0)	67 (38.1)	​	69 (39.2)	​
**Marital status**					
Never married, separated, divorced or widowed	195 (12.2)	54 (27.7)	0.295	80 (41.0)	0.945
Currently married	1408 (87.8)	442 (31.4)	​	574 (40.8)	​
**Smoking history**					
Never and past smoker	1287 (80.3)	431 (33.5)	<0.001	504 (39.2)	0.007
Current smoker	316 (19.7)	65 (20.6)	​	150 (47.5)	​
**Chewing tobacco**					
Never and past user	1218 (76.0)	383 (31.4)	0.438	503 (41.3)	0.470
Current user	385 (24.0)	113 (29.4)	​	151 (39.2)	​
**Body mass index**					
Normal and underweight	693 (43.2)	132 (19.1)	<0.001	281 (40.5)	0.149
Overweight/pre-obesity	591 (36.9)	211 (35.7)	​	256 (43.3)	​
Obese	319 (19.9)	153 (48.0)	​	117 (36.7)	​
**Waist–hip ratio**					
Low	453 (28.3)	90 (19.9)	<0.001	194 (42.8)	0.317
Moderate	313 (19.5)	92 (29.4)	​	117 (37.4)	​
High	837 (52.2)	314 (37.5)	​	343 (41.0)	​
**Diabetes**					
No	1474 (92.0)	425 (28.8)	<0.001	612 (41.5)	0.047
Yes	129 (8.0)	71 (55.0)	​	42 (32.6)	​
**Cardiovascular diseases**					
No	1517 (94.6)	449 (29.6)	<0.001	630 (41.5)	0.012
Yes	86 (5.4)	47 (54.7)	​	24 (27.9)	​
**Other chronic disease**					
No	1454 (90.7)	460 (31.6)	0.060	591 (40.6)	0.699
Yes	149 (9.3)	36 (24.2)	​	63 (42.3)	​
**Family history of hypertension**					
No	1157 (72.2)	313 (27.1)	<0.001	497 (43.0)	0.005
Yes	446 (27.8)	183 (41.0)	​	157 (35.2)	​
**Presence of anxiety**					
No	1,347 (84.0)	392 (29.1)	<0.001	568 (42.2)	0.011
Yes	256 (16.0)	104 (40.6)	​	86 (33.6)	​
**Presence of depression**					
No	1314 (82.0)	377 (28.7)	<0.001	557 (42.4)	0.006
Yes	289 (18.0)	119 (41.2)	​	97 (33.6)	​
**Total**	1603 (100)	496 (30.9)	​	654 (40.8)	​

### Prevalence of hypertension and prehypertension

Table 1 also showed the prevalence of hypertension and prehypertension among rural Bangladeshi adults was 30.9% and 40.8% respectively. Hypertension prevalence was significantly higher among individuals aged 50 years and above (47.3%), while prehypertension was more common among those aged 30 years and younger (44%). Furthermore, hypertension was particularly higher among participants with diabetes (55.0%), CVD (54.7%), and those with obesity (48.0%). Other groups with higher hypertension prevalence included those with a family history of hypertension (41.0%), individuals experiencing symptomatic anxiety (40.6%) and depression (41.2%), participants were retired or students (38.1%), those with no formal education (37.6%), individuals with a high waist-hip ratio (37.5%), and past or non-smokers (33.5%).

In contrast, prehypertension was significantly higher among those who were current smoker (47.5%) and those without diabetes (41.5%) or CVD (41.5%). Prehypertension was also higher among individuals without a family history of hypertension (43.0%) and those with symptomatic anxiety (42.2%) and depression (42.4%) (Table 1).

### Best ML model selection

**Figure 2.**
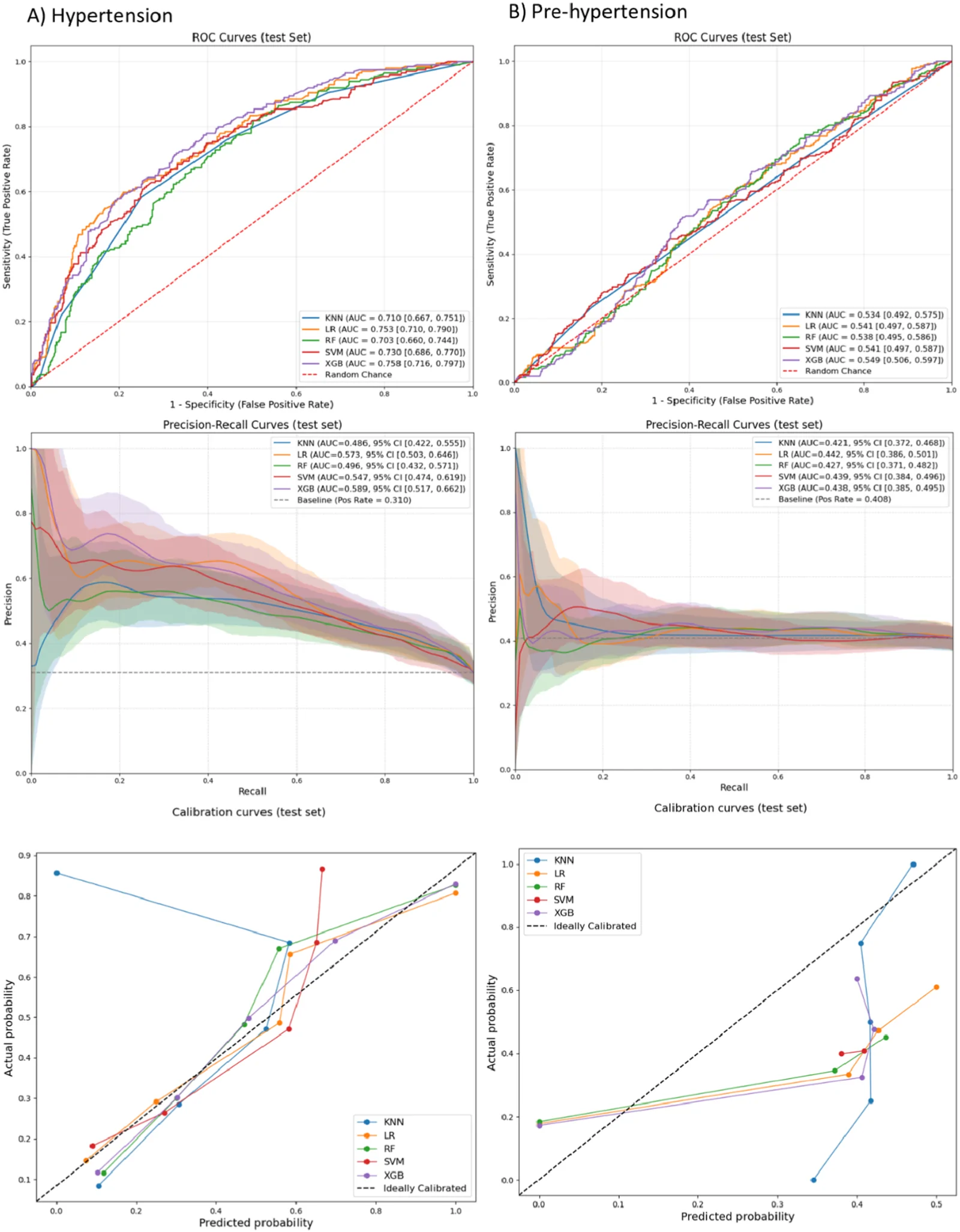
ROC score, PR score and calibration curves for (A) hypertension and (B) prehypertension for validation dataset. Note. • ROC, receiver operating characteristics; PR, precision-recall. • KNN, K-nearest Neighbor; LR, Logistic Regression; RF: Random Forest; SVM, Support Vector Machine. XGB, Extreme Gradient Booster • Higher value for ROC and PR score indicate a better model. For calibration, models’ lines close to ideal line in indicate ideally calibrated.

In predicting hypertension, the XGB model outperformed other ML models across most performance metrics in the validation dataset, achieving an accuracy of 74.3%, an RMSE of 50.7%, and a Brier score of 25.7% (Table 2). The model also demonstrated modest discriminative ability, with an ROC of 75.8% (95% CI: 71.6%–79.7%) and a PR score of 58.9% (95% CI: 51.7%–66.2%) compared to other models. Calibration analysis showed that the XGB model was well-calibrated, with its prediction line closely aligning with the ideal reference line (Figure 2). The XGB model also demonstrated strong performance across additional metrics, achieving a sensitivity of 56.3%, specificity of 79.9%, precision of 68.0%, and an F1 score of 68.0%.

**Table 2.** Models’ performance (test/validation dataset) in predicting hypertension and pre-hypertension.

Models	Performance metrics of models for hypertension						
	Accuracy	RMSE	Sensitivity/recall	Specificity	Precision	F1 score	Brier score
KNN	0.710	0.538	0.347	0.862	0.640	0.610	0.290
LR	0.735	0.515	0.578	0.801	0.690	0.690	0.265
RF	0.701	0.547	0.487	0.772	0.630	0.630	0.299
SVM	0.713	0.535	0.402	0.892	0.700	0.660	0.287
XGB	0.743	0.507	0.563	0.799	0.680	0.680	0.257

Models	Performance metrics of models for pre-hypertension						
	Accuracy	RMSE	Sensitivity/recall	Specificity	Precision	F1 score	Brier score
KNN	0.582	0.646	0.687	0.350	0.520	0.480	0.417
LR	0.592	0.639	0.649	0.426	0.540	0.520	0.408
RF	0.568	0.656	0.622	0.450	0.540	0.520	0.431
SVM	0.590	0.640	0.706	0.321	0.520	0.470	0.410
XGB	0.561	0.663	0.538	0.489	0.510	0.510	0.439

*Note.* KNN, K-Nearest Neighbor; LR, Logistic Regression; RF, Random Forest; SVM, Support Vector Machine; XGB, Extreme Gradient Boosting; RMSE, root mean square error.

For Accuracy, Sensitivity, Specificity, Precision and F1 score, higher values indicate better model performance. For RMSE and Brier score lower values indicate better model performance.

The LR model outperformed other ML models in predicting prehypertension in the validation dataset, achieving better results across most performance metrics (Table 2). It achieved an accuracy of 59.2%, an RMSE of 63.9%, a precision of 54.0%, an F1 score of 52.0%, and a Brier score of 40.8%. Regarding discrimination, the LR model achieved an ROC of 54.1% (95% CI: 49.7%–58.7%), which was slightly lower than that of the XGB model (54.9%, 95% CI: 50.6%–59.7%) (Figure 2). However, the LR model showed a higher PR score of 44.2% (95% CI: 38.6%–50.1%). Among all models, the calibration curve for the LR model indicated slightly better alignment with the ideal reference line. The LR model showed promising performance for other metrics including sensitivity (64.9%), specificity and (42.6%). Furthermore, the performance metrics to train all models were presented in Supplementary Table S5 and Supplementary Figure S2.

### Interpretability of influential factors in the model

**Figure 3.**
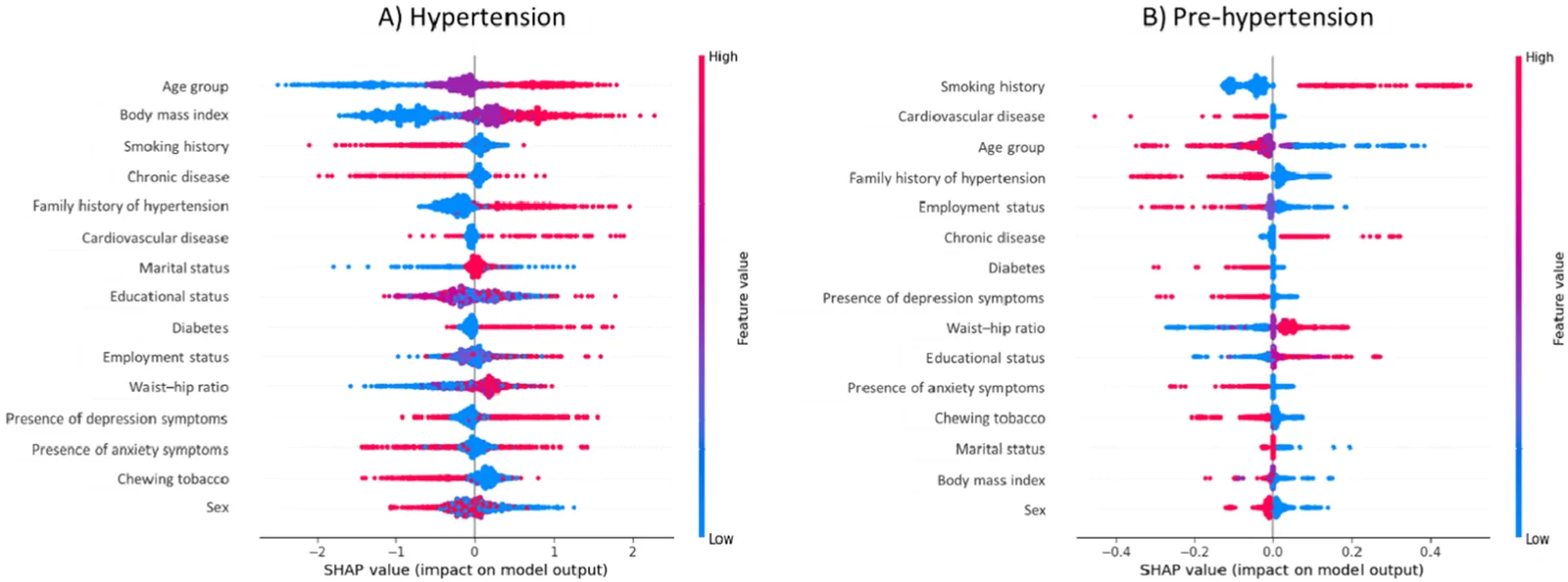
SHAP Beeswarm plots to explain the influential factors related to (A) hypertension and (B) prehypertension. *Note.* SHAP, SHapley Additive exPlanations. Interpretation of the SHAP plot: The SHAP (Shapley Additive Explanations) plot provides a clear visualization of how individual variables influence the model’s predictions. In this plot, the horizontal axis represents SHAP values, where positive values indicate an increased likelihood of the outcome, while negative values indicate a decreased likelihood. Each dot corresponds to an individual observation, and the color of the dot represents the magnitude of the variable, with blue indicating lower values and red indicating higher values. For categorical variables, coded scales are used; for example, in Figure 3(A), age group is coded as 0 for ≤30 years, 1 for 31–50 years, and 2 for ≥50 years. Accordingly, blue dots represent younger individuals, while red dots represent older individuals. The distribution of dots shows that higher age groups (red dots) are predominantly located on the positive side of the axis, indicating that older individuals have a higher likelihood of hypertension. Conversely, lower age groups (blue dots) are mainly positioned on the negative side, suggesting a reduced likelihood of hypertension among younger individuals. Overall, the SHAP plot effectively demonstrates both the direction and strength of each variable’s contribution to the model outcome.

In Figure 3, the most influential factors associated with hypertension were, in order, age, BMI, smoking history, chronic disease, and family history of hypertension. Specifically, individuals of older age, those who were overweight or obese, had a past or no smoking history, reported absence chronic disease, and had a family history of hypertension were more likely to have hypertension.

Similarly, in Figure 3, the most influential factors associated with prehypertension, in order, were current smoking status, absence of CVD, being in a younger or middle-aged group, having no family history of hypertension, and current employment.

## Discussion

This study indicated that the prevalence of hypertension and prehypertension was 30.9% and 40.8%, respectively, among rural adults, which was higher compared to the national level.^1,6^ Furthermore, several community-based studies across different rural areas demonstrate varying prevalence of hypertension (16.0% to 25.3%) and prehypertension (18.7% to 31.9%) in different time periods.^3,14,17^ Given the current status of hypertension in rural Bangladesh, this study utilized sophisticated ML models (KNN, LR, RF, SVM, XGB) based on their promising performance in prior research to predict hypertension.^19,23–25,38^ Findings indicated that the XGB and LR models performed marginally better than random guessing in predicting these conditions. Additionally, several demographics, anthropometric, lifestyle, and clinical factors were identified as influential factors of hypertension and prehypertension.

While a few studies have applied ML methods to predict hypertension in Bangladesh, the use of ML for predicting prehypertension remains unexplored.^23–25^ This study found that the XGB model outperformed others in predicting hypertension, with higher performance metrics including accuracy, RMSE, ROC, PR, Brier score and calibration plot, which aligns with findings from previous studies.^19,25^ Although the LR model has shown better performance in predicting hypertension in various population-based studies,^25,38^ it outperformed other ML models in predicting prehypertension in this study. While previous studies in Bangladesh have primarily relied on traditional regression models to identify influential factors for hypertension and prehypertension,^5,6,14^ this study highlights the potential of ML models to predict these conditions in clinical practice and identify key factors.

Extending the narration, in real-world settings, ML models enable early detection of high-risk individuals, guide resource allocation in low-resource systems, and support policy planning through scenario-based projections. Integration into digital health platforms allows frontline workers to use risk scores for screening and referral, while population-level predictions help identify geographic clusters of risk. However, success depends on data quality, interpretability, and ethical protection; poor or unclear models can undermine trust and accuracy. In this study, the XGB model identified numerous influential factors associated with hypertension, including age as the top-order variable, followed by BMI, smoking history, chronic disease, and family history of hypertension. Older age, being overweight or obese, and a family history of hypertension were the well-established predictors found in different traditional regression and ML-based studies in Bangladesh and other developing countries.^3,10,16,17,23–25,38^ Older age is associated with arterial stiffening and endothelial dysfunction, leading to increased peripheral resistance and hypertension.^43^ Likewise, higher BMI is associated with sodium retention, increased sympathetic activity, insulin resistance, which leads to volume overload and hypertension.^44^ In this study, non-smokers or past-smokers were more prone to become hypertensive. The higher risk of hypertension among non-smokers and former smokers may be due to post-cessation weight gain and metabolic rebound, both of which increase blood pressure. Current smokers often have lower BMI and experience short-term blood pressure reductions from nicotine, which can mask hypertension in measurements.^45,46^ Additionally, reverse causation likely plays a role, as many individuals quit smoking after being diagnosed with hypertension, making former smokers appear at higher risk despite smoking not being protective.^47,48^ The observed pattern may be further explained by cumulative tobacco exposure among former smokers, which causes lasting vascular damage even after cessation, older age, which independently increases blood pressure, and a higher burden of comorbidities such as obesity, diabetes, or cardiovascular disease, all of which elevate hypertension risk as potential residual confounding.^49–51^ This study also showed that the absence of chronic diseases was associated with a higher likelihood of hypertension. The lack of association in this study does not necessarily mean that chronic diseases are unrelated to hypertension in general. It is also possible that these conditions were underreported due to a lack of skilled health care professionals and inadequate diagnostic facilities, or less common in the rural setting, which may have reduced their apparent effect.^52^ Further, the influence of stronger factors such as age, lifestyle, and body weight also could be the reason.

For prehypertension, the LR model found current smoking status, absence of CVD, younger or middle age, no family history of hypertension, and current employment as the most influential factors. Smoking induces inflammation, endothelial dysfunction, and oxidative stress, thereby altering blood pressure regulation.^53^ Similarly, cessation of smoking could resolve these conditions and reduce the risk of hypertension. Furthermore, the absence of CVD and no family history of hypertension was associated with prehypertension, demonstrating that the asymptomatic rural population is often less engaged with the healthcare system, and potentially without a genetic history, they are less likely to be screened and remain undiagnosed. A study conducted among the rural population in southeastern Bangladesh reported that approximately half of the study participants never measured their blood pressure.^54^ Moreover, younger or middle-aged individuals, and employment status were associated with occupation-related stress, a sedentary lifestyle, which further increases the risk of prehypertension.^55^

Despite the launch of the Non-Communicable Disease Control Program (NCDC) in 2002, which was supported by the World Health Organization and the Directorate General of Health Services (DGHS) Bangladesh, the prevalence of hypertension in rural Bangladesh continues to escalate, driven by a shortage of healthcare professionals, limited preventive screening, inconsistent medication supplies, long travel distances, and financial constraints.^29,56,57^ Amid the rising burden of hypertension, this study underscores the need for strong multisectoral collaboration in public health initiatives in rural Bangladesh, including mass screening programs, strengthened primary healthcare, community health education, and trained personnel. These efforts are crucial to improve awareness, screening, and management of hypertension, concentrating focus on the young and middle-aged populations, who are falling through the cracks of the healthcare system. Finally, integrating updated research findings with existing national policies and surveillance systems may improve the detection and management of hypertension.

This study has notable strengths and limitations. Its primary strength lies in the successful application of robust ML methods and in a thorough evaluation of factors associated with both hypertension and prehypertension. Additionally, a standard blood pressure measuring protocol was utilized: BP was measured three times, and the average was used. Furthermore, to ensure the accuracy of locally affordable BP measuring devices, a subset of readings was cross-checked against a clinically certified device, adding an extra layer of quality control. However, no variation in BP measurements was accounted for, and.

Limitations include that the white-coat effects could not be considered due to limitations in obtaining clinical records in a community-based survey setting. The age distribution of the sample population differs from the national average, potentially affecting estimates of hypertension prevalence. The study design is cross-sectional, which limits causal inference. Also, the retrospective, self-reported data introduces recall bias. Geographically, the study was conducted in a single rural area in Bangladesh; although it shares demographics with other rural areas, unique local factors may limit the generalizability of the findings to all of rural Bangladesh.

Additionally, while powerful at identifying patterns, ML models do not provide p-values or beta coefficients to assess variable associations. Overfitting in ML can occur when the sample size is small, as the model may capture noise rather than a generalizable pattern. Notably, the LR model, developed to predict prehypertension, showed low discrimination power (ROC-AUC, 54.1%). This underscores its limitations for direct clinical practice or as a standalone screening tool in its current form, as it could misclassify a large proportion of the population. Despite this, it serves as a crucial benchmark and reflects the challenges of predicting pre-hypertension with common demographics and lifestyle data alone. To avoid complexity, this study did not compare the performance and findings of ML models and traditional LR models. External validation was not conducted due to the unavailability of independent datasets.

## Conclusion

Three out of ten individuals were hypertensive, while two out of five were prehypertensive in rural Bangladesh, which was higher. The XGB was an effective model for predicting hypertension, whereas LR performed slightly better than random chance at predicting prehypertension. Key predictive factors identified across the models included older age, those who were overweight or obese, had a past or no smoking history, reported absence of chronic disease, and had a family history of hypertension for hypertension, whereas current smoking status, absence of CVD, being in a younger or middle-aged group, having no family history of hypertension, and current employment for prehypertension. The study highlights the urgent need for stronger national and regional public health initiatives to improve hypertension detection, awareness, and management in rural Bangladesh. The ML models used in this study demonstrated moderate predictive performance for hypertension and slightly better performance for prehypertension. These findings need cautious interpretation. It is a call for further evaluation of the findings with large, diverse datasets, along with ML applications, to ensure effective public health practice. Furthermore, external validation is necessary to establish the generalizability of findings.

## Supplemental material

Supplemental material - Machine learning approaches to identify influential factors associated with hypertension and prehypertension among rural adults in Bangladesh Supplemental material for Machine learning approaches to identify influential factors associated with hypertension and prehypertension among rural adults in Bangladesh by Md Zahidul Islam, Mohammad Rocky Khan Chowdhury, Zarin Raihana, Farzana Akhter Bornee, Farah Naz Rahman, Shanta Rani Biswas, and Mamunur Rashid in Journal of Public Health Research.

## Data Availability

The data that supports the findings of this study are available from the senior author upon reasonable request.*
