# Supplementary material for: Machine learning approaches to identify influential factors associated with hypertension and prehypertension among rural adults in Bangladesh
Source: J Public Health Res. 2026 Aug 25;15(3):22799036261467444. doi: 10.1177/22799036261467444 (PMC13519169; doi:10.1177/22799036261467444)
Supplement: Supplementary file 1 [file sj-pdf-1-phj-10.1177_22799036261467444.pdf]

Supplementary information

Supplementary Text S1. Recruitment and training workshop for field personnel

We have appointed five interviewers (along with two local support persons) with a minimum of graduate-level education and social science backgrounds, and with previous experience in data collection on an ad hoc basis. A registered medical nurse was appointed and trained to measure anthropometrics and blood pressure. The chief investigator primarily organised a six-day, one-week online (Zoom) workshop to familiarise the interviewers with the study's objective and interview questionnaire. All data collectors and support persons strictly followed a safety process (wearing a face mask, maintaining a physical distance of 1.5 meters, and using hand sanitiser before and after each procedure). Additionally, two support persons have been recruited from the local area of the data collection site. They helped the team navigate the area and build rapport with local residents. Mock interviews were conducted as a pre-test to ensure interviewers could practice relevant techniques and adapt to difficult situations during the main interview.

Supplementary Text S2. Data collection instrument

Data was collected by face-to-face interviews using a validated semi-structured questionnaire. The original questionnaire was in English, which was translated into Bengali for the convenience of local comprehension facilitating data collection. The Bengali version was then converted into English to ensure linguistic and conceptual equivalency. The survey questionnaire had the following sections: participants' demographic and life-style factors; and participants' clinical data, which includes hypertension related questions. As a component of face validity, the research team subjectively evaluated the questionnaires presentation and relevance to ensure the items were pertinent, reasonable, unambiguous, and clear. Additionally, content validity was ensured through a comprehensive literature review, followed by expert panel evaluation in the relevant research field.

Supplementary Text S3. Pilot study

A pilot study was conducted with 24 participants from the selected sampling area to assess the questionnaire's acceptability and feasibility, and to determine the average time required to

complete the survey. After the pilot study, no major modifications were made to the questionnaire, except for a few minor language changes. The pilot study data were not included in the study sample. The questionnaire took approximately 30 min per participant to complete.

Supplementary Text S4. Quality of assurance

To ensure the quality of data collection, we implemented several measures: (i) prior to the survey, the questionnaire underwent pre-testing to identify and address inconsistencies, unclear language, and prolonged administration time. Following appropriate modifications, the questionnaire was finalized; (ii) pre-survey workshop organized for field personnel to outline the purpose of the study, the procedures and potential difficulties related to data collection; (iii) maintained strict monitoring of the data collection and management processes was regularly conducted by one of the local senior investigators; and (iv) utilization of resilient equipment for physical and clinical measurements. The investigators conducted a random consistency check on at least 5% of the interviewed questionnaires.

Supplementary Table S1. Measurements of independent variables.

Variable	Description	Measurement and codes	Scale of measurement
**Demographic variables**			
Age (in years)	Participants age in years during the data collection period	<35 (0), 35-50 (1) and ≥51 (2)	Categorial
Sex	Sex of the participants	Male (0), female (1)	Binary
Educational status	Educational status can be categorized as "educated" when they have completed a minimum of five years of schooling, typically corresponding to the primary (class 1 to 5), secondary (class 6 to 10), and/or higher level. On the other hand, if there are no schooling years completed (0 years of schooling), this categorized them as "no formal education."	No formal education (0), primary (1), secondary (2), Higher (3)	Categorical
Employment status	Participants engaged in economic activity at the time of data collection.	Employed or self-employed (0), Housewife (1), retired or student (2)	Categorical
Marital status	Participant's marital relationship status during the data collection period.	Others (never married, separated, divorced, or widowed) (0), Currently married (1)	Binary
**Lifestyle factors**			
Chewing tobacco	Adaptable behaviours and ways of life like influence the participant's health and well-being.	Past and non-smoker (0), current smoker (1)	Binary
Smoking history	Adaptable behaviours and ways of life like influence the participant's health and well-being	Past and non-smoker (0), current smoker (1)	Binary
Body mass index (BMI)	BMI was derived from the measured values of height and weight. Using WHO's BMI guidelines for the Asian population, BMI has been classified. **Height** was measured using a portable stadiometer (Seca 213 Portable Stadiometer) with light clothing and while barefoot. Study participants were instructed to stand straight against a wall looking forward with loosely hanging arms on their sides. The head plate was put on the crown of the head and a measurement on tape was noted to the nearest 0.5 cm. The measurement was taken twice. If the difference was more than 2 mm, a third measurement was taken. The average of the measurements was recorded as the participant's eight. A third measurement was taken if the difference was more than 2 mm. The average of the measurements was recorded as the participant's height. Further, participants' **weight** was measured using a digital weighing scale (BEURER wellbeing PS 240*)* with light clothing and while barefoot. Participants were instructed to stand still in the center of the machine looking forward with loosely hanging arms on their sides. Weight was recorded to the nearest 0.1 kg.	Underweight (<18.50 kg/m^2) (0), normal (18.50- 22.99 kg/m^2) (1), overweight (23.00-27.49 kg/m^2) (2), and obese (≥27.50 kg/m^2) (3)	Categorical
Waist–hip ratio	The waist–hip ratio is the dimensionless ratio of the circumference of the waist to that of the hips. This was calculated as waist circumference (WC)	Low (0), moderate (1), high (2)	Categorical

	divided by hip circumference (HC) (WC/HC). Optimal cut-off values of waist–hip ratio was measured. The cut-off points are 85 cm for Asian men and 75–80 cm for Asian women and categorised as low, moderate and high. Participants' WC was measured while they were wearing thin clothing (if they were clothed for cultural reasons), on exhalation, midway between the lower rib margin and the anterior superior iliac spine (hip bone), or the narrowest abdominal point. Participants were relaxed with arms hanging loosely by their sides. The tape measure was kept horizontal for a standing measurement. This was done twice. If the measurements differed by more than 2 cm, a third measurement has been taken. The average of the measurements was recorded as the participant's WC. Further, a measurement was taken at the widest circumference around the hip bones so that the tape passes over the greatest protrusion of the gluteal muscles. The tape measure was kept horizontal for a standing measurement. This was done twice. If the measurements differed by more than 2 cm, a third measurement was taken. The hip circumference was recorded to the nearest 0.5 cm.		
**Clinical characteristics**			
Diabetes	Participant living with diabetes mellitus was considered if there was a documented diagnosis and/or medication history.	No (0), yes (1)	Binary
Cardiovascular diseases	Patients had heart attack and stroke received treatment and/or were taking prescribed medication.	No (0), yes (1)	Binary
Other chronic disease	Chronic diseases such as kidney disease, asthma, cancer, arthritis or others were considered to be present if there was a documented diagnosis, medication history, or any past procedures.	No (0), yes (1)	Binary
Family history of hypertension	Any blooded family member had high blood pressure	No (0), yes (1)	Binary
Presence of Anxiety symptoms	The Generalized Anxiety Disorder-7 (GAD-7) scale comprises seven questions. The indicators showed high internal consistency with Cronbach's α being 0.78. Participants were asked to have anxiety symptoms in two weeks prior to the interview using a 4-point Likert scale with options 0 (not at all), 1 (several days), 2 (more than half the days), and 3 (nearly every day). Based on the different cut-off of GAD-7 score, anxiety was categorized as no or minimal anxiety (0-4), mild anxiety (5-9), moderate anxiety (10-14), and severe anxiety (≥15). It was further coded binary as 0 (<5) for no or minimal anxiety and 1 coded for symptomatic anxiety for rest of the categories (score: ≥5).	No (0), yes (1)	Binary
Presence of Depression symptoms	Depression symptoms was assessed using the Patient Health Questionnaire-9 (PHQ-9) scale which consists of nine questions. The indicators demonstrated strong internal consistency, with a Cronbach's α	No (0), yes (1)	Binary

	of 0.73. Participants were asked to have depressive symptoms in two weeks prior to the interview using a 4-point Likert scale with options 0 (not at all), 1 (several days), 2 (more than half the days), and 3 (nearly every day). Adding up scores for individual question makes a total score of 27. Based on the different cut-off of PHQ-9 score, symptomatic depression was categorized as no or minimal depression (0-4), mild depression (5-9), moderate depression (10-14), moderately severe depression (15-19) and severe depression (20-27). Following the previous research, the coding was extended to binary form: 0 (<5) denoted absence or minimal presence of symptomatic depression, while 1 indicated the presence of symptomatic depression for all other categories (score: 5-27).		

Supplementary Table 2. Description of selected machine learning algorithms.

Items	Description
K-nearest neighbour (KNN)	KNN is a non-parametric learning method that classifies a data point based on the majority class of its *k* closest neighbours. It is simple, interpretable, and effective for datasets with well-defined local patterns but can be sensitive to the choice of k and feature scaling.
Logistic Regression (LR)	LR is a supervised machine learning algorithm commonly used for binary and multiclass classification. It estimates the probability of class membership using the sigmoid function and fits model parameters through maximum likelihood estimation.<br><br>LR assumes a linear relationship between the input features and the log-odds of the outcome. To reduce the risk of overfitting, regularization techniques such as L1 (lasso) and L2 (ridge) are often applied.
Random Forest (RF)	RF is an ensemble machine learning algorithm that builds multiple decision trees using randomly selected subsets of data and features. The final prediction is made by aggregating the outputs of these individual trees, typically through majority voting for classification or averaging for regression.<br><br>RF is known for its robustness, high accuracy, and ability to model complex, non-linear relationships. It also reduces the risk of overfitting compared to single decision trees and provides useful measures of feature importance, making it a powerful and versatile tool for both classification and regression tasks.
Support Vector Machine (SVM)	SVM is a supervised learning algorithm that identifies the optimal hyperplane to separate classes in feature space. It is effective for high-dimensional datasets, can model non-linear relationships using kernel functions, and is widely used in both classification and regression tasks.
Extreme Gradient Booster (XGB)	XGB is a high-performance machine learning algorithm used for both classification and regression tasks. As an advanced implementation of the gradient boosting framework, XGB builds decision trees sequentially, with each tree correcting the errors of the previous ones to improve overall model accuracy.<br><br>XGB is known for its speed, scalability, and strong predictive performance. Key features include built-in regularization to reduce overfitting, efficient handling of missing data, and the ability to process large datasets rapidly. Its versatility and robust performance have made it a widely adopted tool in many real-world applications.

Supplementary Table 3. Model optimization**.**

Items	Hyperparameter optimization	Cross-validation (k-fold)	Parameters	Thresholds
K-nearest neighbours (KNN)	Grid search	10 folds	n_neighbors	0.5
Logistic Regression (LR)	Grid search	10 folds	random_state, C, penalty, solver, scoring	0.5
Random Forest (RF)	Grid search	10 folds	n_estimators, min_samples_split, max_features	0.5
Support Vector Machine (SVM)			'C', kernel, probability, gamma, scoring, n_jobs	0.5
Extreme Gradient Booster (XGB)	Grid search	10 folds	random_state, objective, alpha, gamme, eval_metrics, eta, learning_rate, max_delta_step, nthread, n_estimators, max_depth, min_child_weight, colsample_bytree, colsample_bylevel, scoring	0.5

Supplementary Table 4. Definitions of performance metrics.

True positive	A true positive occurs when the model correctly predicts an instance as belonging to the positive class
True negative	A true negative is an outcome where the model correctly predicts an instance as belonging to the negative class
False positive	A false positive is an outcome where the model incorrectly predicts an instance as belonging to the positive class
False negative	A false negative is an outcome where the model incorrectly predicts an instance as belonging to the negative class.
Accuracy	Model accuracy is the proportion of correct predictions made by the model, calculated as the sum of true positives and true negatives divided by the total number of predictions:<br>Accuracy = (True positives + True negatives) / (True positives + True negatives + False positives + False negatives).
Root Mean Square Error (RMSE)	RMSE= $\sqrt{\frac{1}{n}\sum_{i=1}^{n}(y_i - \hat{y}_i)^2}$<br>(RMSE) measures the average magnitude of prediction errors, giving greater weight to larger errors. Lower RMSE values indicate higher model accuracy and better predictive performance.
Sensitivity/recall	Sensitivity measures a test’s ability to correctly identify individuals with the disease. A highly sensitive test produces few false negatives, meaning it is effective at minimizing missed cases.<br>Sensitivity = true positive /(false negative + true positive)
Specificity	Specificity measures the model’s ability to correctly identify individuals without the condition, reflecting the proportion of true negatives among all those who are actually negative.<br>Specificity = true negative / (true negative + false positive)
Precision	Precision, also known as Positive Predictive Value (PPV) represents the

	proportion of correctly predicted positive cases among all cases predicted as positive.<br>Precision = True positive/(True positive + False positive)
F1 score	The F1 score is the harmonic mean of precision and recall, providing a balanced measure of a model's accuracy. The F1 score is particularly useful when the costs of false positives and false negatives are similar. The F1 score is especially important for imbalanced datasets, where relying solely on accuracy can be misleading.<br>F1=2*(Precision*recall)/(Precision + Recall)<br>Interpretation:<br>• F1 = 1: Perfect precision and recall, indicating an ideal model.<br>• F1 = 0: Either precision or recall is zero, representing the worst performance.<br>• Higher F1 Score: Indicate better performance, with the model accurately identifying positive cases while minimizing errors.<br>• Lower F1 Score → Indicates poor performance, with difficulties in handling false positives or false negatives.
Receiver Operating Characteristics (ROC)	The Receiver Operating Characteristic (ROC) curve plots sensitivity against 1 – specificity, and the Area Under the Curve (AUC).<br>ROC= $\int_{x=0}^{1}[Sensitivity\ \{(1-Specificity)^{-1}\ (x)\}]dx$
Precision-Recall (PR) curve	The PR curve is used to assess the performance of classification models, especially when dealing with imbalanced datasets. It plots Precision (the accuracy of positive predictions) against Recall (the ability to identify all positive cases) across different decision thresholds. High values of both Precision and Recall indicate a strong model, while low values suggest poor performance. The area under the PR curve (AUC-PR) provides a summary measure of model effectiveness, with higher scores reflecting better performance.
Brier score	The Brier Score measures the accuracy of probabilistic predictions by quantifying the difference between predicted probabilities and actual binary outcomes. The Brier Score ranges from 0 to 1, where 0 indicates perfect accuracy and calibration, and 1 reflects completely inaccurate predictions. Lower scores represent better model calibration and predictive accuracy.<br>Brier score = 1/N$\sum_{i=1}^{N}(f_i - o_i)^2$<br>Where:<br>• N = Total number of predictions<br>• f_i = Predicted probability for instance iii<br>• o_i = Actual outcome (1 for positive, 0 for negative)
Calibration plot	A calibration plot illustrates how well predicted probabilities correspond to actual observed outcomes. The x-axis shows the predicted probabilities, while the y-axis represents the observed proportion of events. A perfect calibration is depicted by a diagonal line, where predictions match outcomes exactly. Deviations from this line indicate miscalibration: points above the line suggest the model is underestimating risk, whereas points below indicate overestimation.

Supplementary Table S5. Models' performance (train/derivation dataset) in predicting hypertension and pre-hypertension.

Models	Performance metrices of models for hypertension						
	**Accuracy**	**RMSE**	**Sensitivity/recall**	**Specificity**	**Precision**	**F1 score**	**Brier score**
**KNN**	0.749	0.501	0.407	0.917	0.730	0.680	0.251
**LR**	0.735	0.515	0.512	0.803	0.660	0.660	0.265
**RF**	0.881	0.344	0.801	0.902	0.850	0.850	0.119
**SVM**	0.735	0.515	0.418	0.916	0.730	0.680	0.265
**XGB**	0.752	0.498	0.562	0.818	0.690	0.690	0.248
**Models**	**Performance metrices of models for pre-hypertension**						
	**Accuracy**	**RMSE**	**Sensitivity/recall**	**Specificity**	**Precision**	**F1 score**	**Brier score**
**KNN**	0.718	0.532	0.952	0.550	0.770	0.710	0.283
**LR**	0.594	0.637	0.714	0.445	0.580	0.550	0.405
**RF**	0.642	0.598	0.760	0.554	0.660	0.640	0.358
**SVM**	0.658	0.585	0.883	0.654	0.760	0.750	0.342
**XGB**	0.631	0.608	0.676	0.580	0.620	0.620	0.369

Note: KNN, K-Nearest Neighbor; LR, Logistic Regression; RF, Random Forest; SVM, Support Vector Machine; XGB, Extreme Gradient Boosting; RMSE, root mean square error

For Accuracy, Sensitivity, Specificity, Precision and F1 score, higher values indicate better model performance. For RMSE and Brier score lower values indicate better model performance

Supplementary Figure S1. Sampling technique.

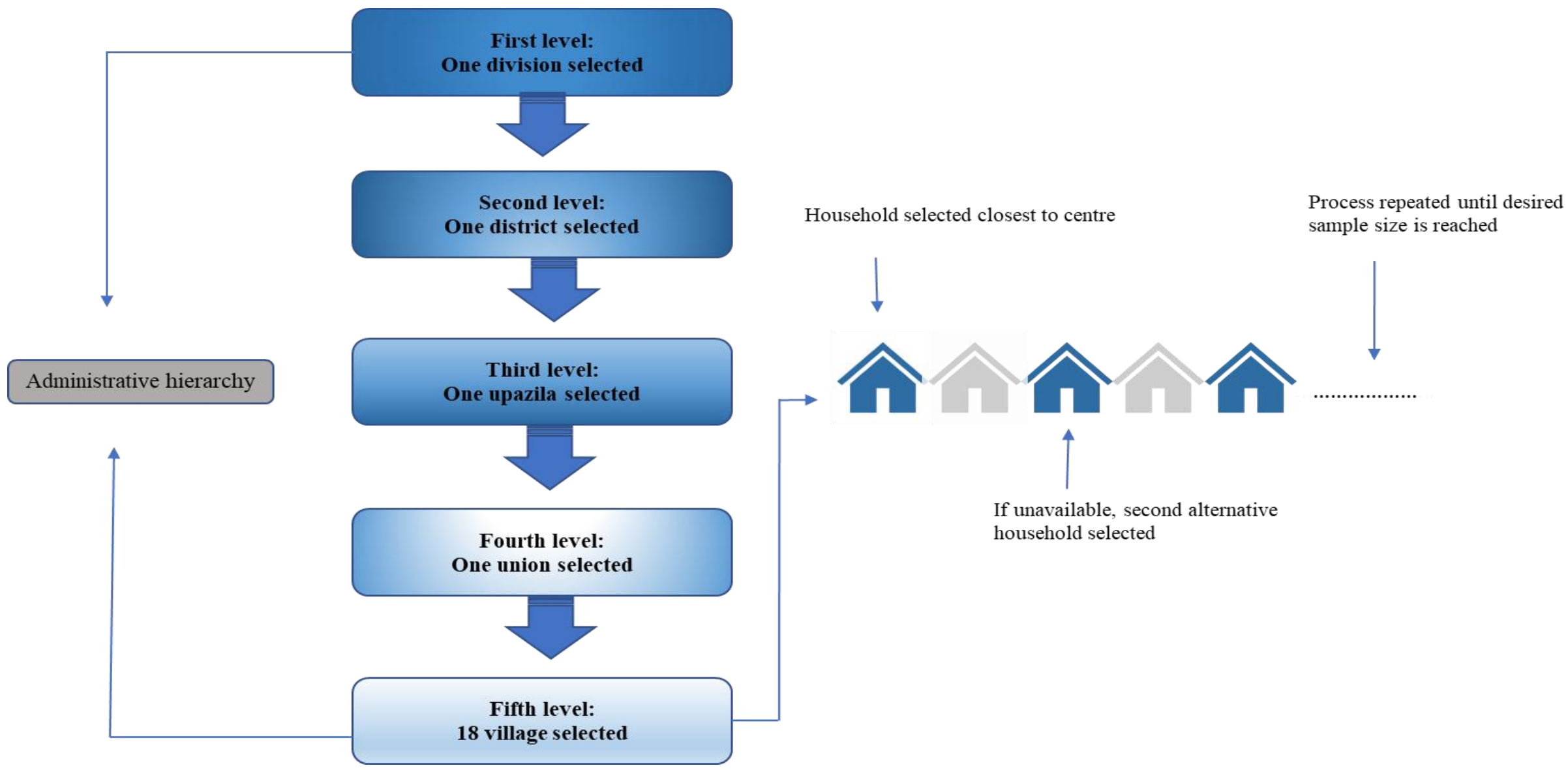

Supplementary Figure S2. ROC score, PR score and calibration curves for A) hypertension and B) prehypertension for train dataset.

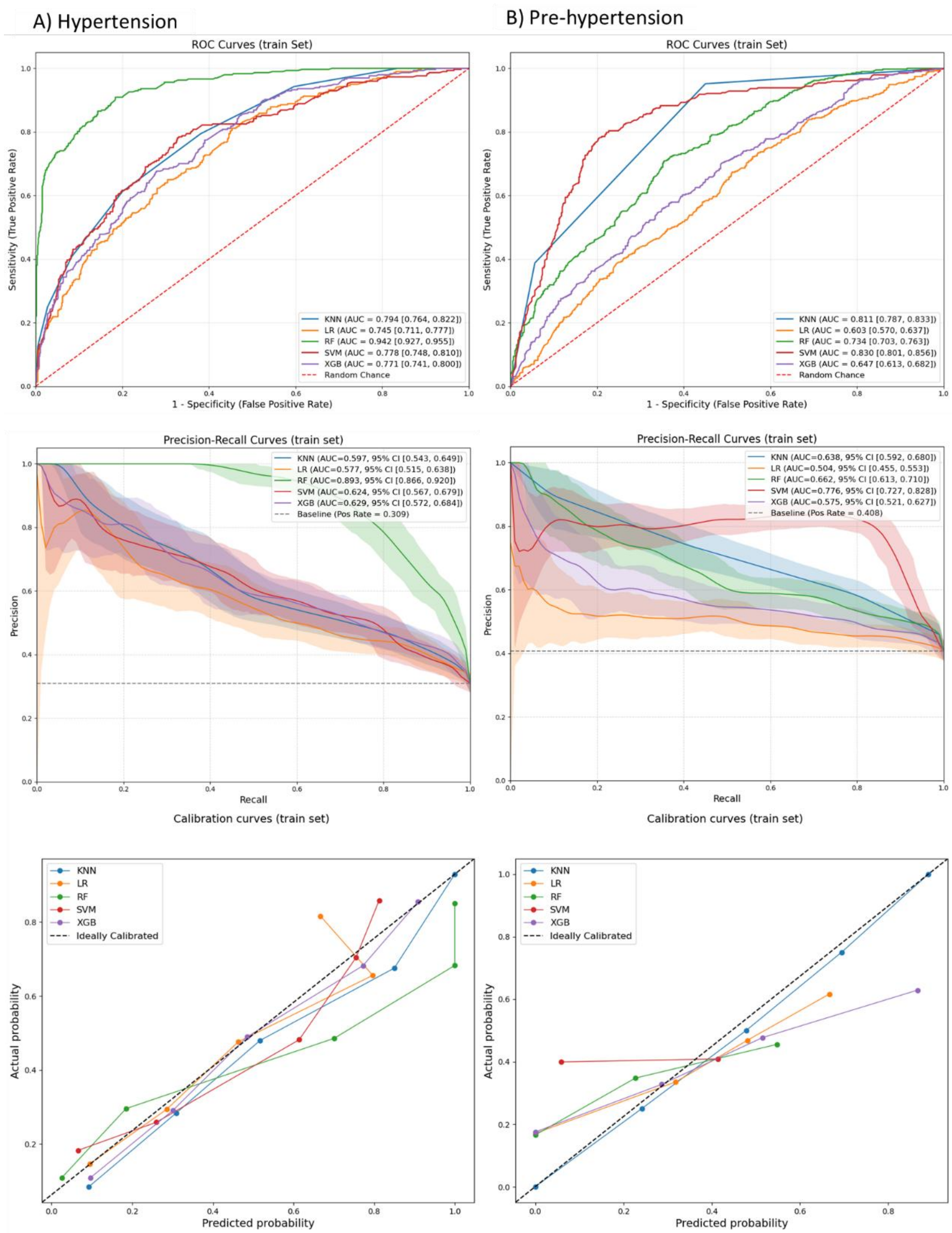

Note:

- ROC, receiver operating characteristics; PR, precision-recall
- KNN, K-nearest Neighbor; LR, Logistic Regression; RF: Random Forest; SVM, Support Vector Machine. XGB, Extreme Gradient Booster
- Higher value for ROC and PR score indicate a better model. For calibration, models' lines close to ideal line in indicate ideally calibrated.
